# Association between metabolic score for insulin resistance and lower limb pain among the elderly: a cross-sectional study

**DOI:** 10.3389/fendo.2025.1530067

**Published:** 2025-06-24

**Authors:** Yanfen Hu, Junxia Yu, Zhenjie Xu, Yan Gao, Yang Li, Lingxia Li

**Affiliations:** ^1^ Department of Geriatric Endocrinology, Metabolism and Respiratory Medicine (the Cadre Ward), The Second Affiliated Hospital of Xi’an Jiaotong University, Xi’an, Shaanxi, China; ^2^ Jianjiyue Biomedical Research Center, Xi’an, Shaanxi, China

**Keywords:** lower limb pain, insulin resistance, metabolic syndrome, metabolic score for insulin resistance, cross-sectional study

## Abstract

**Purpose:**

This study aims to investigate the association between the metabolic score for insulin resistance (Mets-IR) and lower limb pain (LLP) among the elderly.

**Methods:**

Baseline and follow-up data were collected from the China Health and Retirement Longitudinal Study (CHARLS) database in 2011 and 2018, respectively. Multivariable logistic regression analysis and restricted cubic spline were performed to explored the relationship between Mets-IR and LLP. Subgroup analysis was also conducted.

**Results:**

A total of 4983 participants were included in the study, among which 3350 (67.2%) suffered from LLP. Logistic regression analysis confirmed a statistically significant relationship between Mets-IR and LLP risk (*p* < 0.001). Restricted cubic spline plots indicated that the association between Mets-IR and LLP is not nonlinear. Both subgroup and interaction analyses hinted at the influence of variation in sleep duration on this correlation.

**Conclusions:**

The findings indicate a positive correlation between Mets-IR and LLP risk, which is influenced reciprocally by sleep duration.

## 1 Introduction

Lower limb pain (LLP) is a prevalent condition that encompasses discomfort in the hip, knee, ankle, and foot (1). The Centers for Disease Control and Prevention (CDC) has identified LLP as a significant source of chronic pain among adults (2). This type of pain is particularly common among the elderly, with many seniors experiencing discomfort in multiple lower limb joints simultaneously (3). LLP is often associated with activities that exert stress on the lower limbs, such as frequent jumping, bouncing, and sudden strenuous movements (4). As research into LLP has advanced, several key risk factors have been identified. These include advancing age, gender, physical inactivity, heart disease, chronic metabolic disorders (e.g., kidney disease), and obesity (5, 6). Moreover, metabolic disorders have been shown to contribute to joint pain in the limbs (7). Notably, the severity of knee pain is closely linked to metabolic syndrome and its components, with central obesity being a primary mediator of severe knee pain (8).

Insulin resistance (IR) is a state characterized by reduced sensitivity and responsiveness to the actions of insulin, often occurring several years prior to the onset of diabetes (9). Studies have shown that hyperinsulinemia induced by IR accelerates the production of fatty acids, impedes the normal function of insulin, and triggers early atherosclerosis and abnormal blood pressure (10). The Metabolic Score for Insulin Resistance (Mets-IR) is a quantitative tool used to evaluate the degree of IR in individuals by integrating multiple metabolic indicators associated with IR. It combines parameters such as fasting plasma glucose (FPG), triglycerides (TG), high-density lipoprotein cholesterol (HDL-C), and body mass index (BMI) to reflect the overall state of metabolic disturbances in the body (11). Mets-IR is a commonly used clinical surrogate marker that can effectively identify individuals at high risk of IR-related pathological changes (11). A cohort study conducted on the Chinese population found that, compared to the TG/HDL-C ratio and the triglyceride-glucose index (TyG), Mets-IR demonstrates stronger predictive ability for cardiovascular diseases (12). Nevertheless, the relationship between Mets-IR and LLP remains underexplored, particularly with regard to the potential association between LLP and Mets-IR in older adults.

The objective of this study was to examine the relationship between these two variables. A better understanding of the factors influencing LLP may be gained by assessing them using the Mets-IR. This would facilitate a more detailed study of LLP-related diseases.

## 2 Materials and methods

### 2.1 Study design and data source

The data for this study originated from the 2011 iteration of the China Health and Retirement Longitudinal Study (CHARLS). CHARLS, a nationally representative longitudinal survey in China, focuses on adults aged 45 and older and their spouses, encompassing evaluations of their social, economic, and health conditions. Conducted between June 2011 and March 2012, CHARLS 2011 enrolled 17,705 participants from 450 communities spanning 28 provinces. The CHARLS was ethically approved by the institutional review board at Peking University (IRB00001052-11015). All participants signed an informed consent form before taking part in the survey (13, 14).

### 2.2 Participants

**Figure 1:**
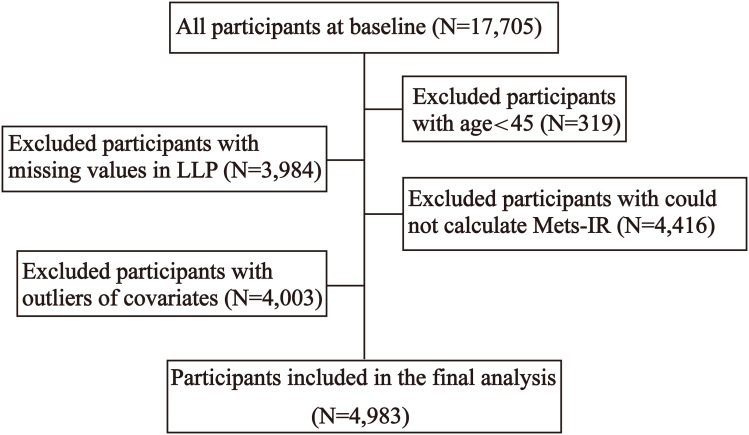
Flow chart of participants selection. Flow chart for selecting the study population from the database of CHARLS.

The data for this study were derived from baseline data collected in 2011 and follow-up data collected in 2018. The exclusion criteria were as follows: 1) age < 45; 2) missing values in LLP; 3) outliers in covariates; 4) could not calculate Mets-IR. Ultimately, 4,983 participants were included in the study (**Figure 1**).

### 2.3 Outcomes

LLP was assessed based on responses to the following question: Are you often troubled by pain in any part of your body? If the participants answered in the affirmative, the following question was: on what part of your body do you feel pain?

### 2.4 Assessment of Mets-IR

The Mets-IR was calculated according to a previous study (9).

BMI = weight (kg) ÷ the square of height (m^2^);

TG/HDL-C = TG (mg/dL) ÷ HDL-C (mg/dL);

TyG = Ln [TG (mg/dL) × FPG (mg/dL) ÷2];

Mets-IR = Ln [(2×FPG (mg/dL) + TG (mg/dL)] × BMI (kg/m^2^) ÷ Ln [HDL-C (mg/dL)].

### 2.5 Variables

The demographic variables included age, gender, education, marital status and location. Smoking and drinking status, sleep duration, and BMI (categorized as underweight [< 18.5 kg/m^2^], normal [18.5-25.0 kg/m^2^], overweight [25.0-30.0 kg/m^2^], obesity [≥ 30.0 kg/m^2^]) were used as variables for health-related behaviors. Blood biomarkers including TG, HDL-C, and FPG.

### 2.6 Statistical analysis

Categorical variables are expressed as percentages, whereas normally distributed continuous variables are expressed as means and standard deviations (SD). One-way ANOVA or Chi-square tests were employed to ascertain the characteristics and risk of developing LLP subsequent to the grouping of Mets-IR by quartiles (Q1, Q2, Q3, and Q4). Three logistic models were constructed using Mets-IR as a categorical variable (quartiles) to estimate the 95% confidence intervals (CI) and the odds ratio (OR) for LLP. The three models investigated the correlation between Mets-IR and LLP, encompassing an unadjusted model (Model 1), an adjusted model incorporating age, gender, education, location and marital status (Model 2), and a further adjusted model that considered smoking status, drinking status, BMI and sleep duration based on Model 2 (Model 3). All statistical analyses were conducted using R 4.4.1, and restricted cubic splines were performed with the “rms” package. A two-tailed p < 0.05 was considered statistically significant.

## 3 Results

### 3.1 Baseline characteristics

Participant characteristics are presented in **Table 1**. A total of 4,983 individuals were included in this study, with females accounting for 60.7% and males 39.3%. Among them, 67.2% suffered from LLP. In the LLP group, the proportion of females was higher than that of males (64.4% vs. 35.6%). Most participants in this group were aged 45–60 years (59.2%). The proportion of married individuals was higher than that of unmarried (83.3% vs. 16.7%). Their educational level was generally low, with a large majority having an education level below primary (92.4%). Most of them located in villages (94.4%), and a significant proportion had short sleep durations, with over half sleeping less than 6 h (55.9%). Notably, the Mets-IR value was higher in the LLP group compared to the non-LLP group (36.1 vs. 35.3, *p* < 0.05). Additionally, medication use had no significant impact on the incidence of LLP, as there were no notable differences between the two groups in this regard.

**Table 1:** The basic characteristics of participants at baseline.

Variables	Overall	Non-LLP	LLP	*p*
N (%)	4983 (100)	1633 (32.8)	3350 (67.2)	
Age (year, %)				<0.001
45-60	3075 (61.7)	1092 (66.9)	1983 (59.2)	
60-90	1905 (38.2)	539 (33.0)	1366 (40.8)	
>90	3 (0.1)	2 (0.1)	1 (0.0)	
Gender (%)				<0.001
Female	3023 (60.7)	865 (53.0)	2158 (64.4)	
Male	1960 (39.3)	768 (47.0)	1192 (35.6)	
Education (%)				<0.001
College+	48 (1.0)	22 (1.3)	26 (0.8)	
High school	390 (7.8)	162 (9.9)	228 (6.8)	
Primary-	4545 (91.2)	1449 (88.7)	3096 (92.4)	
Marital (%)				0.002
Married	4208 (84.4)	1417 (86.8)	2791 (83.3)	
unmarried	775 (15.6)	216 (13.2)	559 (16.7)	
Location (%)				0.657
City	287 (5.8)	98 (6.0)	189 (5.6)	
Village	4695 (94.2)	1535 (94.0)	3160 (94.4)	
Take anti-hypertensive drugs (%)				0.730
Yes	963 (75.8)	258 (75.0)	705 (76.1)	
No	307 (24.2)	86 (25.0)	221 (23.9)	
Take anti-diabetic drugs (%)				0.949
Yes	186 (64.4)	49 (65.3)	137 (64.0)	
No	103 (35.6)	26 (34.7)	77 (36.0)	
Take anti-tumor drugs (%)				0.924
Yes	24 (49.0)	8 (53.3)	16 (47.1)	
No	25 (51.0)	7 (46.7)	18 (52.9)	
Take anti-stroke drugs (%)				0.730
Yes	963 (75.8)	258 (75.0)	705 (76.1)	
No	307 (24.2)	86 (25.0)	221 (23.9)	
Taking anti-psychotic drugs (%)				0.192
Yes	48 (39.7)	15 (51.7)	33 (35.9)	
No	73 (60.3)	14 (48.3)	59 (64.1)	
Smoking (%)				<0.001
Non-smoker	3240 (65.0)	967 (59.2)	2273 (67.9)	
Ex-smoker	1743 (35.0)	666 (40.8)	1077 (32.1)	
Frequency of drinking (%)				<0.001
≥1/per month	1114 (22.4)	438 (26.8)	676 (20.2)	
<1/per month	375 (7.5)	121 (7.4)	254 (7.6)	
None of those	3494 (70.1)	1074 (65.8)	2420 (72.2)	
Sleep duration (h, %)				<0.001
<6	2677 (53.7)	804 (49.2)	1873 (55.9)	
6-9	1949 (39.1)	706 (43.2)	1243 (37.1)	
>9	357 (7.2)	123 (7.5)	234 (7.0)	
BMI (kg/m^2^)				<0.001
<18.5	330 (6.6)	108 (6.6)	222 (6.6)	
18.5-25.0	3049 (61.2)	1072 (65.6)	1977 (59.0)	
25.0-30.0	1334 (26.8)	385 (23.6)	949 (28.3)	
≥30.0	270 (5.4)	68 (4.2)	202 (6.0)	
TG (mg/dL, mean, SD)	134.5 (110.8)	132.5 (115.3)	135.5 (108.6)	0.374
HDL-C (mg/dL, mean, SD)	51.5 (15.2)	51.4 (15.4)	51.6 (15.2)	0.676
FPG (mg/dL, mean, SD)	109.5 (33.8)	107.1 (27.0)	110.7 (36.6)	<0.001
TyG	8.7 (0.7)	8.7 (0.7)	8.7 (0.7)	0.004
TG/HDL-C	3.3 (5.9)	3.3 (5.4)	3.4 (6.2)	0.666
Mets-IR	35.8 (8.6)	35.3 (8.3)	36.1 (8.8)	0.002

Data were summarized as mean (95% confidence intervals) or percentage (95% confidence intervals) according to their data type. LLP, lower limb pain; BMI, body mass index; TG, triglycerides; HDL-C, high-density lipoprotein cholesterol; FPG, fasting plasma glucose; Mets-IR, metabolic score for insulin resistance.

**Table 2** presents the characteristics of participants categorized based on the quartiles of Mets-IR. The Mets-IR quartiles are specifically defined as follows: Q1 (< 29.84), Q2 (29.84-34.54), Q3 (34.54-40.40), and Q4 (> 40.40). Compared to the Q1 group, participants in the Q4 group are predominantly aged between 45–60 years (65.5%), female (67.6%), married (87.6%), mostly located in villages (92.4%), with lower educational levels (89.6%), and a higher proportion sleeping less than 6 h (51.1%). Additionally, the majority of participants in the Q4 group have a BMI within the range of 25.0-30.0 kg/m² (63.2%). Furthermore, when comparing the Q1 and Q4 groups, there is a gradual increase in the proportion of participants with LLP (65.2% vs. 71.3%), as well as an increase in the proportion of participants taking antihypertensive and hypoglycemic drugs, with statistically significant differences (*p* < 0.05).

**Table 2:** Weighted baseline characteristics of participants by quartiles of baseline Mets-IR.

Variables	Overall	Q1	Q2	Q3	Q4	*p*
N (%)	4983	1246	1246	1245	1246	
Age (year, %)						<0.001
45-60	3075 (61.7)	671 (53.9)	773 (62.0)	815 (65.5)	816 (65.5)	
60-90	1905 (38.2)	574 (46.1)	472 (37.9)	430 (34.5)	429 (34.4)	
>90	3 (0.1)	1 (0.1)	1 (0.1)	0 (0.0)	1 (0.1)	
Gender (%)						<0.001
Female	3023 (60.7)	674 (54.1)	706 (56.7)	801 (64.3)	842 (67.6)	
Male	1960 (39.3)	572 (45.9)	540 (43.3)	444 (35.7)	404 (32.4)	
Education (%)						<0.001
College+	48 (1.0)	0 (0.3)	11 (0.9)	17 (1.4)	16 (1.3)	
High school	390 (7.8)	69 (5.5)	90 (7.2)	118 (9.5)	113 (9.1)	
Primary-	4545 (91.2)	1473 (94.1)	1145(82.9)	1110 (89.2)	1117(89.6)	
Marital (%)						<0.001
Married	4208 (84.4)	1016 (81.5)	1033(82.9)	1068 (85.8)	1091(87.6)	
Unmarried	775 (15.6)	230 (18.5)	213(17.1)	177 (14.2)	155 (12.4)	
Location (%)						<0.001
City	287 (5.8)	36 (2.9)	60 (4.8)	96 (7.7)	95 (7.6)	
Village	4695 (94.2)	1210 (97.1)	1186(95.2)	1148 (92.3)	1151(92.4)	
Take anti-hypertensive drugs (%)						<0.001
Yes	963 (75.8)	111 (64.9)	175 (71.4)	245 (77.5)	432 (80.3)	
No	307 (24.2)	60 (35.1)	70 (28.6)	71 (22.5)	106 (19.7)	
Take anti-diabetic drugs (%)						0.003
Yes	186 (64.4)	9 (40.9)	24 (63.2)	39 (58.2)	114 (70.4)	
No	103 (35.6)	13 (59.1)	14 (36.8)	28 (41.8)	48 (29.6)	
Take anti-tumor drugs (%)						0.781
Yes	24 (49.0)	6 (46.2)	3 (42.9)	9 (60.0)	6 (42.9)	
No	25 (51.0)	7 (53.8)	4 (57.1)	6 (40.0)	8 (57.1)	
Take anti-stroke drugs (%)						0.212
Yes	66 (65.3)	8 (72.7)	13 (65.0)	14 (50.0)	31 (73.8)	
No	35 (34.7)	3 (27.3)	7 (35.0)	14 (50.0)	11 (26.2)	
Taking anti-psychotic drugs (%)						0.345
Yes	48 (39.7)	7 (28.0)	14 (38.9)	16 (51.6)	11 (37.9)	
No	73 (60.3)	18 (72.0)	22 (61.1)	15 (48.4)	18 (62.1)	
Smoking (%)						<0.001
Non-smoker	3240 (65.0)	708 (56.8)	788 (63.2)	858 (68.9)	886 (71.1)	
Ex-smoker	1743 (35.0)	538 (43.2)	458 (36.8)	387 (31.1)	360 (28.9)	
Frequency of drinking (%)						<0.001
≥1/per month	1114 (22.4)	352 (28.3)	291 (23.4)	266 (21.4)	205 (16.5)	
<1/per month	375 (7.5)	95 (7.6)	106 (8.5)	92 (704)	82 (6.6)	
None of those	3494 (70.1)	799(64.1)	849 (68.1)	887 (71.2)	959 (77.0)	
Sleep duration (h, %)						0.103
<6	2677 (53.7)	691 (55.5)	686 (55.1)	659 (52.9)	641 (51.4)	
6-9	1949 (39.1)	477 (38.3)	471 (37.8)	480 (38.6)	521 (41.8)	
>9	357 (7.2)	78 (6.3)	89 (7.1)	106 (8.5)	84 (6.7)	
BMI (kg/m^2^)						<0.001
<18.5	330 (6.6)	318 (25.5)	11 (0.9)	1 (0.1)	0 (0.0)	
18.5-25.0	3049 (61.2)	927 (74.4)	1180 (94.7)	748 (60.1)	194 (15.6)	
25.0-30.0	1334 (26.8)	1 (0.1)	55 (4.4)	490 (39.4)	788 (63.2)	
≥30.0	270 (5.4)	0 (0.0)	0 (0.0)	6 (0.5)	264 (21.2)	
TG (mg/dL, mean, SD)	134.5 (110.8)	83.7 (35.0)	105.8 (51.6)	133.7 (65.2)	214.8(176.5)	<0.001
HDL (mg/dL, mean, SD)	51.5 (15.2)	64.8 (15.4)	54.4 (11.6)	47.6 (10.2)	39.2 (10.1)	<0.001
FPG (mg/dL, mean, SD)	109.5 (33.8)	99.4 (17.1)	104.6 (24.8)	109.3 (28.5)	124.8 (49.9)	<0.001
TyG	8.7 (0.7)	8.2 (0.4)	8.5 (0.5)	8.8 (0.5)	9.3 (0.7)	<0.001
TG/HDL-C	3.3 (5.9)	1.4 (0.8)	2.1 (1.3)	3.0 (1.8)	6.9 (10.8)	<0.001
LLP (%)						<0.05
Yes	3350 (67.2)	812 (65.2)	814 (65.3)	835 (67.1)	889 (71.3)	
No	1633 (32.8)	434 (34.8)	432 (34.7)	410 (32.9)	357 (28.7)	

Data were summarized as mean (95% confidence intervals) or percentage (95% confidence intervals) according to their data type. BMI, body mass index; TG, triglycerides; HDL-C, high-density lipoprotein cholesterol; FPG, fasting plasma glucose; Mets-IR, metabolic score for insulin resistance.

### 3.2 Association between Mets-IR and LLP

The data indicate a link between Mets-IR and the risk of LLP (**Table 3**). In the unadjusted Model 1, individuals in the Q2, Q3, and Q4 Mets-IR groups had ORs of 1.00 (95% CI: 0.97-1.04), 1.02 (95% CI: 0.98-1.06), and 1.06 (95% CI: 1.03-1.10) for developing LLP, respectively, compared to those in the Q1 group. After adjusting for age, gender, education, location, and marital status in Model 2, the risk of LLP increased by 7% in the highest Mets-IR quartile compared to the lowest (OR: 1.07, 95% CI: 1.03-1.11, *p* < 0.001). Both Model 1 and Model 2 showed statistically significant trends (*p* < 0.001). Model 3 further adjusted for smoking status, drinking status, BMI, and sleep duration based on Model 2, with fully adjusted ORs and 95% CIs of 1.00 (0.96-1.04), 1.00 (0.96-1.05), and 1.03 (0.97-1.09) for Q2, Q3, and Q4, respectively, compared with Model 1, and with *p* = 0.25 for the trend test.

**Table 3:** Association of Mets-IR with lower limb pain in different models among all participants.

Predictor	Model 1	*p*	Model 2	*p*	Model 3	*p*
Mets-IR per IQR	1.03 (1.01-1.04)	<0.05	1.03 (1.01-1.05)	<0.001	1.00 (0.97-1.04)	0.80
Quartiles of Mets						
Q1	Ref		Ref		Ref	
Q2	1.00 (0.97-1.04)	0.93	1.01 (0.97-1.05)	0.59	1.00 (0.96-1.04)	0.86
Q3	1.02 (0.98-1.06)	0.31	1.03 (0.99-1.06)	0.19	1.00 (0.96-1.05)	0.85
Q4	1.06 (1.03-1.10)	<0.001	1.07 (1.03-1.11)	<0.001	1.03 (0.97-1.09)	0.30
*p* for trend		<0.001		<0.001		0.25

Model 1: no covariables were adjusted; Model 2: adjusted for age, gender, marital status, location, and education level; Model 3: adjusted for age, gender, marital status, location, and education level, smoking status, drinking status, BMI, and sleep duration.

### 3.3 Restricted cubic spline regression

**Figure 2:**
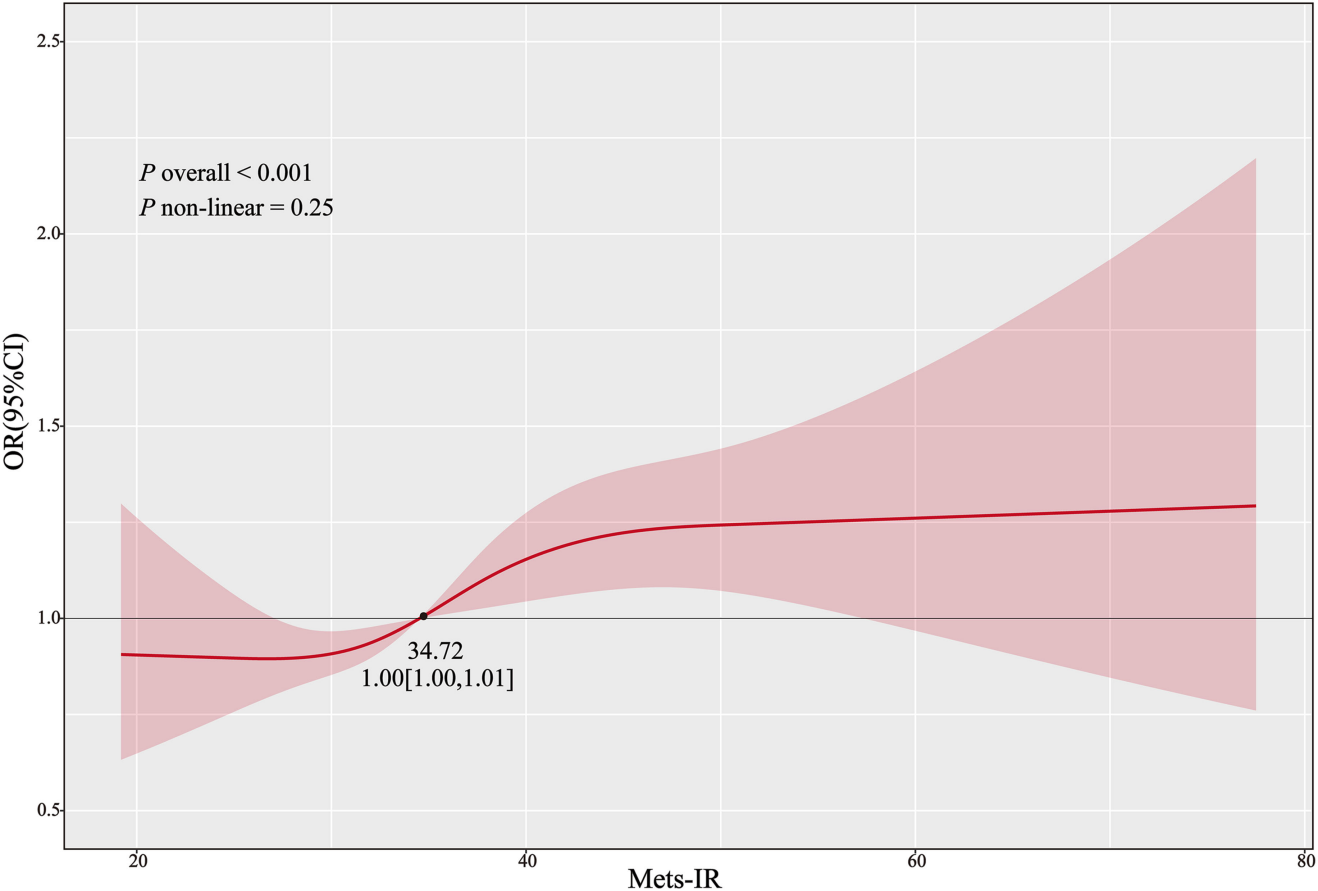
RCS of the association between Mets-IR and the risk of LLP. Restricted cubic spline curves for the association between Mets-IR and LLP risk. The model was adjusted for age, gender, education level, location, and marital status.

Restricted cubic splines were employed to visualize and examine the dose-response relationship between Mets-IR and LLP. As illustrated in **Figure 2**, after adjusting for multiple variables, the p for non-linearity between Mets-IR and LLP = 0.25, the p for the overall was < 0.001. The p for non-linearity tests whether the non-linear term in the model is statistically significant, whereas the p for the overall tests the significance of the entire model. The results indicate that there is no significant non-linear relationship between Mets-IR and LLP. Furthermore, the risk of developing LLP gradually increases after the Mets-IR value > 34.72, and then remains relatively stable.

### 3.4 Subgroup analysis

**Figure 3:**
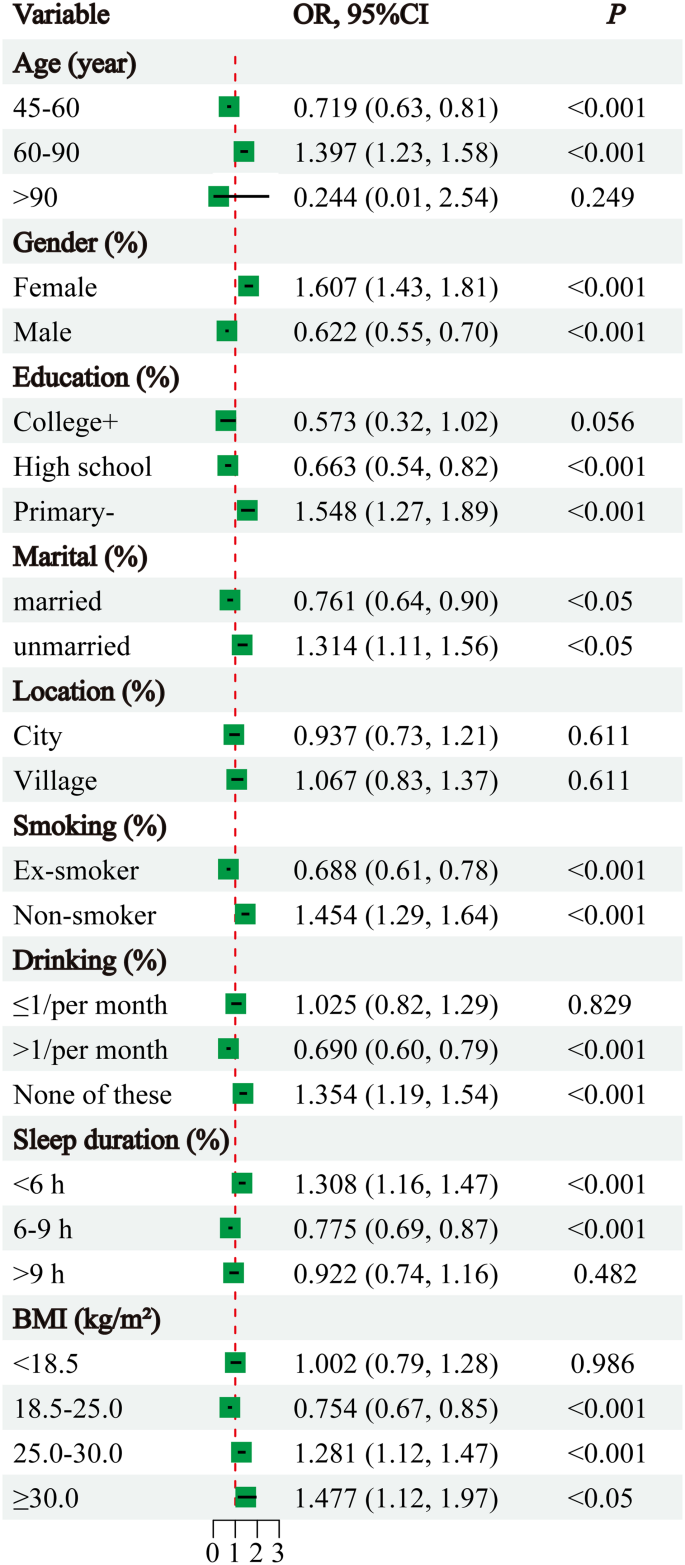
Subgroup analysis. Forest plot of stratified analysis of the relationship between Mets-IR and LLP risk.

**Figure 4:**
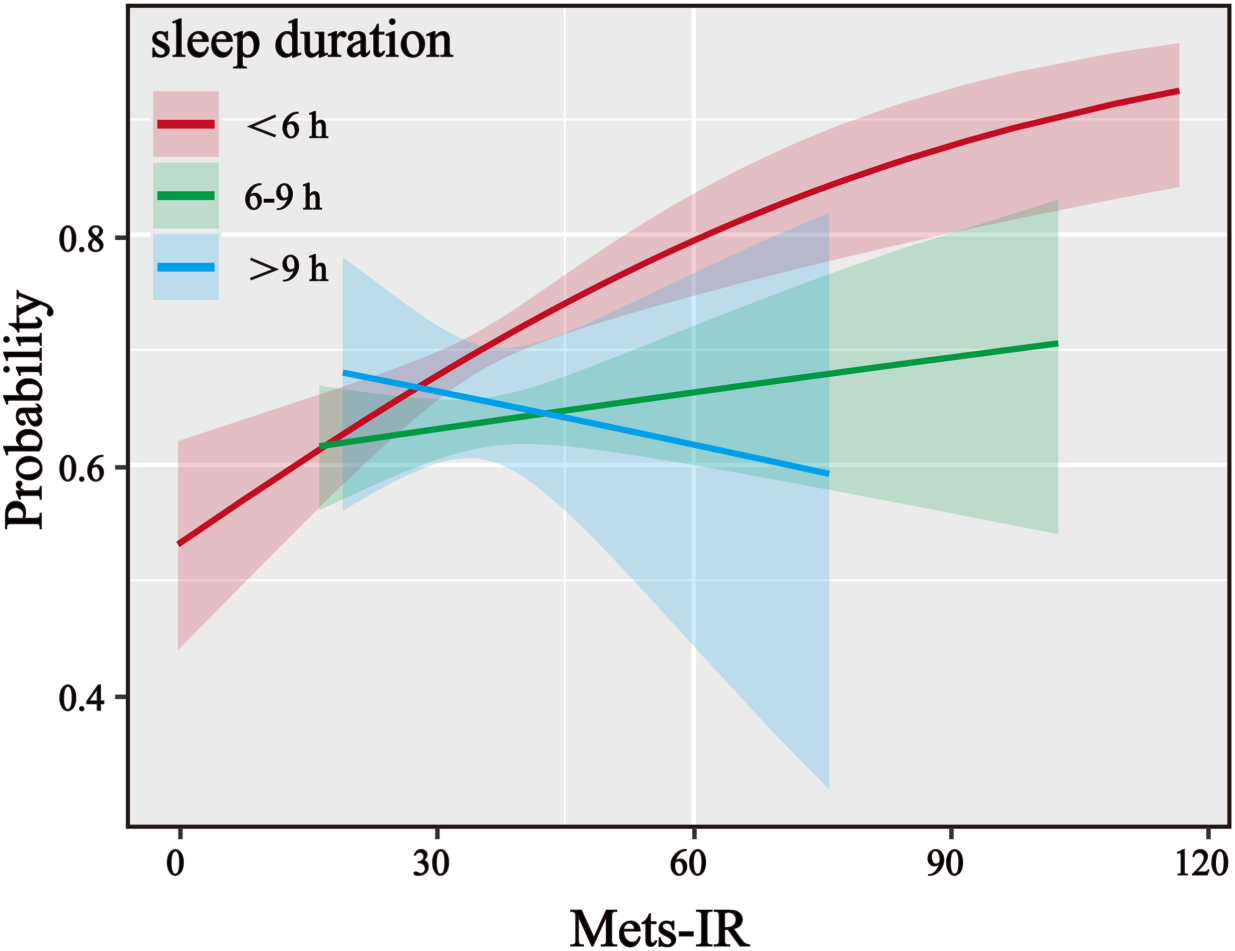
Logistic regression interaction. Logistic regression interaction fitting stratified by sleep duration to confirm the positive correlation between Mets-IR and LLP interacted across cohort characteristics.

To investigate whether there are differences in the impact of Mets-IR on the risk of developing LLP across different subgroups, the data were grouped based on the characteristics of the study participants. The results revealed that age, gender, education level, marital status, smoking status, drinking status, sleep duration, and BMI were all significantly associated with the relationship between Mets-IR and the risk of LLP (**Figure 3**). Further exploration was conducted to examine the interaction effects between different subgroups and the association between Mets-IR and LLP risk. The findings indicated that there were univariate interaction effects between Mets-IR and LLP risk with respect to sleep duration (**Figure 4**).

## 4 Discussion

This study utilized data from the 2011 and 2018 nationwide surveys of the CHARLS database for analysis, aiming to explore the association between Mets-IR and LLP. The results revealed significant differences between the non-LLP group and the LLP group in factors such as age, gender, educational level, marital status, sleep duration, smoking status, drinking status, and BMI. The value of Mets-IR was influenced by age, gender, educational level, marital status, BMI, and the use of antihypertensive and hypoglycemic drugs. This study indicates a significant association between Mets-IR and the risk of LLP.

LLP often occurs in the elderly and has a significant impact on their daily lives (15, 16). Results from a longitudinal study based on Korean elderly individuals showed that 61% of the participants reported experiencing musculoskeletal-related pain. The study found that pain in the legs, knees, and ankles was significantly associated with impaired ability to perform daily activities (17). In addition, physical activity levels, back pain, amputation, and psychological factors also have a significant impact on the risk of developing LLP (18). IR is defined as a reduced response of the body to the physiological effects of insulin, typically caused by a combination of genetic predisposition and adverse environmental factors (19). Research indicates that IR leads to disturbances in glucose and lipid metabolism, triggers chronic inflammation, and increases the risk of cardiovascular diseases (20). This study found that higher Mets-IR values are associated with an increased risk of LLP. This association may be attributed to inflammatory responses. Elevated levels of inflammatory factors, such as tumor necrosis factor-α (TNF-α) and interleukin-6 (IL-6), can lead to vascular endothelial dysfunction and neuroinflammation (21). These inflammatory factors enter the bloodstream, initiating a systemic inflammatory response. They can directly act on leg tissues, activating inflammatory signaling pathways, promoting the infiltration of inflammatory cells, and releasing more inflammatory mediators, ultimately leading to pain (22). Similarly, LLP is often accompanied by inflammation, and reduced physical activity can exacerbate this inflammatory state. Chronic inflammation can lead to elevated levels of pro-inflammatory factors, which in turn interfere with insulin signaling pathways and reduce insulin sensitivity (23). In addition, IR is often accompanied by hyperglycemia and dyslipidemia (abnormal lipid metabolism). These metabolic disturbances may lead to peripheral neuropathy, manifesting as leg pain, numbness, and tingling sensations (24). Hyperglycemia can lead to the accumulation of advanced glycation end-products (AGEs). These substances are capable of damaging nerve fibers, thereby contributing to the development of LLP (25).

Subgroup analysis results indicate an interaction between Mets-IR and LLP in relation to sleep duration. Previous studies have shown that insufficient sleep duration can increase the risk of LLP (26). Among elderly individuals, those experiencing more severe sleep disorders are at a higher risk of developing LLP (27). Insufficient sleep or poor sleep quality can activate the hypothalamic-pituitary-adrenal (HPA) axis, leading to elevated cortisol levels (28). Cortisol can regulate inflammatory responses in the short term, but if it remains at a high level for a prolonged period, it may lead to dysfunction of the body’s immune system, further exacerbating inflammatory reactions and thereby increasing the risk of LLP (22, 29, 30). Sleep duration is also closely linked to insulin sensitivity. Research indicates that insufficient sleep or poor sleep quality can lead to decreased insulin sensitivity, which in turn exacerbates IR (31). Sleep duration also influences pain perception and pain threshold. Insufficient sleep or poor sleep quality may lead to an increased sensitivity to pain (32). This study hypothesizes that when sleep duration is short, IR worsens, and pain perception simultaneously increases. This dual impact may make the association between Mets-IR and LLP even more pronounced.

Although this study has yielded some noteworthy observations, it still has certain limitations. 1) Insufficient data precision. Regarding LLP, the frequency and duration of pain, as well as whether it is neuropathic or rheumatic pain, were not specified. Due to these data limitations, the findings of this study should be considered preliminary explorations. 2) This study is a cross-sectional one, and thus, it is impossible to infer causality. 3) The relatively small sample size limits the generalizability of the results. In future research, it is necessary to further validate this association in multiple ethnic populations. Additionally, more precise data and longitudinal studies are required to verify the association and infer causality.

## 5 Conclusions

In summary, this study suggests that there is a significant association between the Mets-IR and the risk of LLP. Additionally, sleep duration interacts with this relationship. Improving insulin sensitivity and maintaining an appropriate sleep duration may be important and effective measures for preventing the occurrence of LLP.

## Data Availability

Publicly available datasets were analyzed in this study. The data sets used and analyzed in this study are available from the CHARLS (http://charls.pku.edu.cn), a nationally representative longitudinal survey conducted by Institute of Social Science Survey, Peking University.
